# Skeletal Muscle Microbiopsies in Children and Adults—Tolerability, Sample Yield, and Analyzability

**DOI:** 10.1002/mus.70161

**Published:** 2026-01-30

**Authors:** Emil Rydell Högelin, Sebastian Edman, Paulo R. Jannig, Axel Löfgren, Kajsa Thulin, Piotr Michno, Jessica Norrbom, Björn A. Alkner, Ferdinand von Walden, Lotta Fornander

**Affiliations:** ^1^ Department of Medicine and Geriatrics Region Jönköping County Eksjö Sweden; ^2^ Department of Biomedical and Clinical Sciences Linköping University Linköping Sweden; ^3^ Division of Pediatric Neurology, Department of Women's and Children's Health Karolinska Institute Stockholm Sweden; ^4^ Molecular Muscle Physiology and Pathophysiology Group, Department of Physiology and Pharmacology Karolinska Institute Stockholm Sweden; ^5^ Department of Physiology, Nutrition and Biomechanics The Swedish School of Sport and Health Sciences Stockholm Sweden; ^6^ Department of Orthopaedic Surgery Region Östergötland Norrköping Sweden; ^7^ Department of Orthopaedic Surgery Region Jönköping County Eksjö Sweden; ^8^ Department of Orthopaedic Surgery Region Jönköping County Jönköping Sweden; ^9^ Molecular Exercise Physiology Group, Department of Physiology and Pharmacology Karolinska Institutet Stockholm Sweden

**Keywords:** microbiopsies, muscle biopsies, pain, RNA sequencing, visual analog scale

## Abstract

**Introduction/Aims:**

Traditional methods of sampling skeletal muscle tissue are invasive. This study aimed to evaluate a sub‐millimeter core‐biopsy (microbiopsy) as a potentially more tolerable method, with further regard to tissue yield and analyzability of RNA expression.

**Methods:**

Children (9–13 years, *n* = 11) and adults (18–50 years, *n* = 16) were recruited. Microbiopsy and venipuncture were performed, with prior application of local anesthesia cream. Additionally, adults underwent a Bergström muscle biopsy, with infiltrative local anesthesia. Pain was rated using the visual analog scale (VAS), reported as medians (95% CI). Microbiopsy samples were freeze‐dried and weighed. To evaluate RNA sequencing performance at low tissue sample weights, a six‐step incremental tissue ladder (10–500 μg) was analyzed.

**Results:**

Children rated venipunctures and microbiopsies low, at VAS = 0.1 (0.0–0.6) and 1.6 (0.9–3.9), respectively. Microbiopsy pain ratings were slightly higher than venipuncture, *p* < 0.001. Pain ratings in adults were 0.0 (0.0–0.5), 1.8 (1.3–2.4), 2.9 (2.4–3.8), and 2.7 (2.2–3.8) for venipuncture, microbiopsy, Bergström biopsy, and infiltrative local anesthesia, respectively. Microbiopsy was rated less painful than Bergström biopsy and local anesthesia (*p* < 0.05). Children did not rate microbiopsy more painful than adults (*p* = 0.82). Microbiopsies yielded on average 303 (SD 121.8) μg. RNA sequencing detected similar transcriptomic signatures across the tissue ladder.

**Discussion:**

The generally low pain ratings for the microbiopsy procedure support its use as a tolerable method of acquiring skeletal muscle samples in both children and adults. It represents a less painful alternative to Bergström biopsies while still rendering adequate material for RNA sequencing.

**Abbreviations**
PCA: principal component analysis
VAS: visual analogue scale

## 1 Introduction

Multiple biopsy techniques are widely used diagnostically in science and medicine, including: Bergström biopsy [1], conchotome [2], and open technique [3]. These techniques are all well‐proven in acquiring sufficient material for analysis. However, the techniques are all invasive, limiting the inclusion of potential participants. Data on the perceived pain of the different techniques is sparse. There are multiple scales aimed at providing a quantitative measurement of pain [4, 5, 6, 7]. Perceived pain following a Bergström biopsy has been reported to range from four to six using a modified Borg scale (1 = no pain to 10 = worst possible pain) in eleven adult subjects [8]. However, traditional techniques for tissue sampling are likely less tolerable for children, making the procedure particularly rare in a nonclinical setting [9].

Microbiopsies have been described as a less invasive [10] and less painful [8] way of acquiring biological material. A standard definition of microbiopsy is lacking; some include a < 1 mm needle, equaling gage 20 or greater [11]. However, studies also refer to needles as large as 16 gage as microbiopsy [8]. One important point of differentiation between protocols is the need for dermal incision and infiltrative local anesthetics. To perform the dermal incision needed for a Bergström biopsy, administration of infiltrative subcutaneous local anesthetics is generally needed. This is also reported for thicker needle microbiopsy [8]. Hence, a potential advantage of the microbiopsy technique is that the low‐diameter needle, combined with no need for dermal incision, would alleviate the need for infiltrative local anesthesia and lead to a reduced risk of skin scarring.

Multiple studies have previously proven the feasibility of the microbiopsy technique for acquiring muscle tissue in adults [8, 12, 13, 14]. In assessing feasibility, it is important to consider the required material yield to reliably perform analysis that corresponds to the means of acquisition. Guescini and coworkers showed that biopsies from the vastus lateralis acquired by 22 gage fine needle aspiration technique yielded sufficient material for analysis of selected mRNA targets using tandem RT‐PCR [14]. While many underlying analytic principles are shared between established molecular methods such as PCR and newer, next‐generation sequencing methods, it is still unclear whether the microbiopsy technique can provide enough sample yield to support the generation of wider‐encompassing data, such as transcriptomics or proteomics.

This proof‐of‐concept study aimed to evaluate the tolerability of microbiopsy compared to commonly used venipuncture with respect to perceived pain in both children and adults. A comparison of the perceived pain from the traditional Bergström biopsy technique was also performed in the adult group. Additionally, we report on the amount of material resulting from the microbiopsy procedure and assess the analytic possibility of RNA sequencing at input volumes corresponding to the observed microbiopsy sample yield.

## 2 Method

### 2.1 General Design

Two groups of participants were recruited: adults and children. Both groups underwent microbiopsy and venipuncture in a randomized order. Lastly, the adult participants also underwent a Bergström biopsy. Directly following microbiopsy and venipuncture, respectively, the participants rated their pain sensation using the VAS, a 10‐centimeter‐long sliding scale in which zero corresponds to no pain and 10 to the worst imaginable pain. Participants were instructed to slide a ruler according to their perceived pain, and values were reported in centimeters. Additionally, adults also rated the perceived pain following Bergström biopsy and the preceding local anesthesia administration. All participants received a cutaneous anesthetic agent, lidocaine and prilocaine, applied on a patch (EMLA, Aspen Nordic, Ballerup, Denmark) 1 h prior to microbiopsy and venipuncture. The adults underwent three repeated microbiopsies to secure additional material for inter‐test evaluations; however, only the VAS grading from the first microbiopsy is presented in this study. If any of the procedures were unsuccessful in terms of material yield, repeated attempts were conducted. In that case, the VAS rating following the repeated biopsy was used. The perceived pain was graded by the participant directly following each procedure. Since auditory stimulation has been shown to decrease procedural pain [15], all participants were allowed to pick the music of their choice during the procedure. Inclusion criteria for children were aged nine through 13 years, attending Swedish middle school recruited by informational advertisements, able to give informed assent, and whose legal guardians provided informed written consent. The adult group were 18–50 years old and recruited from advertisements at large workplaces and by word of mouth. For both groups, the exclusion criteria were as follows: current or previous history of myopathy, other neuromuscular diseases, current infection, or anticoagulation therapy or hematological diseases rendering skeletal muscle biopsy unsuitable. This study was performed in accordance with the declaration of Helsinki. Approval was granted by the Swedish Ethical Review Authority (Dnr 2021‐04307).

### 2.2 Microbiopsy

The microbiopsy was performed using a reusable biopsy instrument (Magnum Reusable Core Biopsy Instrument, BD, Franklin Lakes, NJ) and gage 20 single‐use needles (Magnum Needle, BD). An appropriate length of needle was chosen, varying between 10, 13, or 16 cm, depending on the thigh size. The biopsies were performed on the anterolateral portion of the mid‐thigh aiming for the vastus lateralis, and lateralization was randomized. No additional anesthetic was applied beyond the cutaneous plaster. The skin was disinfected using chlorhexidine gluconate and alcohol skin antiseptic. The needle was used to pierce both the skin and the muscle fascia. The procedure was performed using clean but nonsterile gloves; however, the single‐use needle was treated as sterile, and care was taken not to contaminate the device. Following the procedure, a surgical plaster was placed over the incision site, and an elastic bandage was applied, with tension, for 20 min. Microbiopsies and Bergström biopsies were performed by B.A.A. and L.F., both consultant orthopedic surgeons with considerable experience performing biopsies.

### 2.3 Venipuncture

Venipuncture was performed by an experienced nurse using a 23 gage needle (BD Vacutainer, BD, Franklin Lakes, NJ). The site of puncture was the median cubital vein. Cutaneous local anesthetic (EMLA) was applied on both arms in case of failure to acquire blood at the first site. If initial sampling was unsuccessful, the VAS rating was acquired for the successful procedure.

### 2.4 Bergström Biopsy

A conventional skeletal muscle biopsy was performed using the Bergström needle (Stille AB, Torshälla, Sweden) [1], with a suction‐modified Bergström technique [16]. The Bergström biopsy was performed in the contralateral thigh to the one in which the microbiopsy was performed, at the midpoint of the thigh. Approximately 5–10 mL of infiltrative local anesthetic, 10 mg/mL Carbocain (Aspen Nordic) was applied subcutaneously. Following a minimum of 10 min, patient sensitivity was tested by lightly pressing a scalpel against the skin. If this produced any painful sensation, additional time and/or additional anesthetic was administered. When the skin was considered numb to pain, an approximately 1 cm‐long incision was made to facilitate entry of the Bergström needle. The Bergström needle was then used to pierce the muscle fascia and obtain the skeletal muscle sample. Following the biopsy procedure, the wound was closed with Steri‐Strips (3 M, Maplewood, Minnesota) and enclosed with a surgical plaster. An elastic bandage was applied with tension for 20 min following the biopsy.

### 2.5 Material Handling, Freeze‐Drying, and Weighing

All the microbiopsy samples and parts of the Bergström biopsy were frozen in liquid nitrogen and subsequently stored on dry ice until final storage in a −80°C freezer. The microbiopsy samples and part of the Bergström biopsy samples were then freeze‐dried overnight. To assess sample yield from the microbiopsy procedure, the freeze‐dried microbiopsy samples were subsequently weighed on an ultra‐micro balance with readability down to 0.1 μg (Cubis MCA2.7S –2S00–M, Sartorius Lab Instruments GmbH & Co, Göttingen, Germany). Freeze‐dried muscle samples from the Bergström biopsy were initially dissected free from blood and connective tissue under a stereomicroscope. The freeze‐dried Bergström biopsy muscle samples were then pulverized.

### 2.6 RNA‐Sequencing Ladder

To evaluate the analyzability of the sample yield from the microbiopsy procedure, we created a muscle tissue input ladder for next‐generation sequencing analyses (i.e., RNA sequencing; RNA seq). Pulverized freeze‐dried muscle tissue from the vastus lateralis of four participants was utilized. The muscle samples were each divided into separate parts weighing 500, 250, 100, 50, 25, and 10 μg. RNA was extracted from the samples using the filter colon‐based RNeasy Fibrous Tissue Mini Kit (Qiagen, Hilden, Germany). Library preparation of mRNA was performed using SMARTer Total RNA‐Seq Pico Input Mammalian (Takara Bio, San Jose, California, USA), followed by RNA sequencing by an Illumina NovaSeq 6000 (150 bp pair‐end sequencing; SciLife Lab, Solna, Sweden). The RNA seq data were processed using Kallisto for pseudoalignment and gene quantification. Gene count data were imported into DESeq2 for normalization and unsupervised principal component analysis. A gene was considered detected if it had nonzero counts in a particular sample. Presented gene numbers include protein coding and nonprotein coding genes.

### 2.7 Statistics

Data were initially investigated and characterized by descriptive statistics. Statistical analysis was performed in SPSS Statistics for Windows, Version 29.0.2.0 (IBM, Armonk, New York, USA), and figures were generated using GraphPad Prism version 10.0.0 for Windows (GraphPad Software, Boston, Massachusetts, USA). The VAS rating in both adults and children was analyzed using the Wilcoxon signed‐rank test. In the group of adults, a post hoc correction was performed using the Bonferroni method to adjust the alpha level. Both adjusted (*p*_*a*_) and unadjusted (*p*_*r*_) values are reported. The perceived pain between children and adults during microbiopsy was analyzed using the Mann–Whitney *U* test. Gene count was analyzed using repeated‐measures ANOVA with a Bonferroni post hoc analysis. One missing value (V3, 25 μg; Figure 2B,C) was replaced using interpolation (V1 10 μg Gene count/Mean 10 μg Gene count × Mean 25 μg Gene count). Alpha of ≤ 0.05 was deemed to be significant.

## 3 Results

**FIGURE 1:**
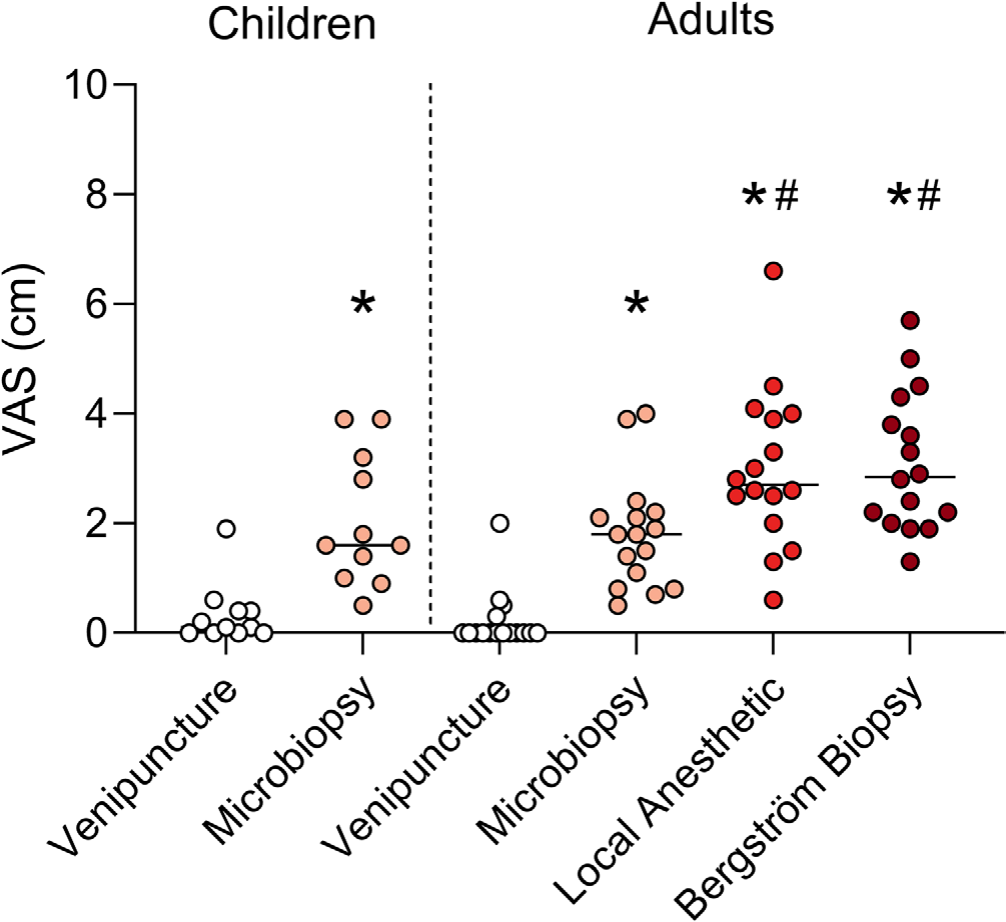
Visual analog scale (VAS) ratings of perceived pain in children and adult participants. Median values are indicated by the horizontal line. Dots represent individual values. * = *p* < 0.05 versus venipuncture, # = *p* < 0.05 versus microbiopsy.

A total of 11 children and 16 adults were included in this study. The mean (SD) ages of adults and children were 11.7 (0.9) and 34.4 (7.7) years, respectively. All participants underwent the procedures without complications, and no one elected to drop out following primary inclusion. No adverse events occurred during this study. In the group of children, VAS (reported as median (95% CI)) was rated higher during microbiopsy 1.6 (1.2–2.9) centimeters compared to venipuncture 0.3 (0.0–0.7); see Figure 1 (*p* = 0.01). For adults, VAS rating was 0.0 (0.0–0.5), 1.8 (1.3–2.3), 2.8 (2.4–3.8), 2.7 (2.2–3.7) centimeters for venipuncture, microbiopsy, Bergström biopsy, and local anesthetic prior to Bergström biopsy, respectively. Similar to the children, the adults rated microbiopsy as more painful than venipuncture *p*_*a*_ = 0.003 (*p*_*r*_ < 0.001). However, both local anesthetic and Bergström biopsy were perceived as more painful than venipuncture, *p*_*a*_ = 0.003 (*p*_*r*_ < 0.001) and *p*_*a*_ = 0.003 (*p*_*r*_ < 0.001), respectively. The microbiopsy was perceived as less painful than local anesthetic and Bergström biopsy, *p*_*a*_ = 0.049 (*p*_*r*_ = 0.008) and *p*_*a*_ = 0.005 (*p*_*r*_ < 0.001), respectively. Bergström biopsies and the local anesthetic procedures were rated as equally painful *p*_*a*_ = 1 (*p*_*r*_ = 0.798). There were no significant differences between children and adults regarding perceived pain in response to venipuncture, *p* = 0.012 or microbiopsy, *p* = 0.82.

**FIGURE 2:**
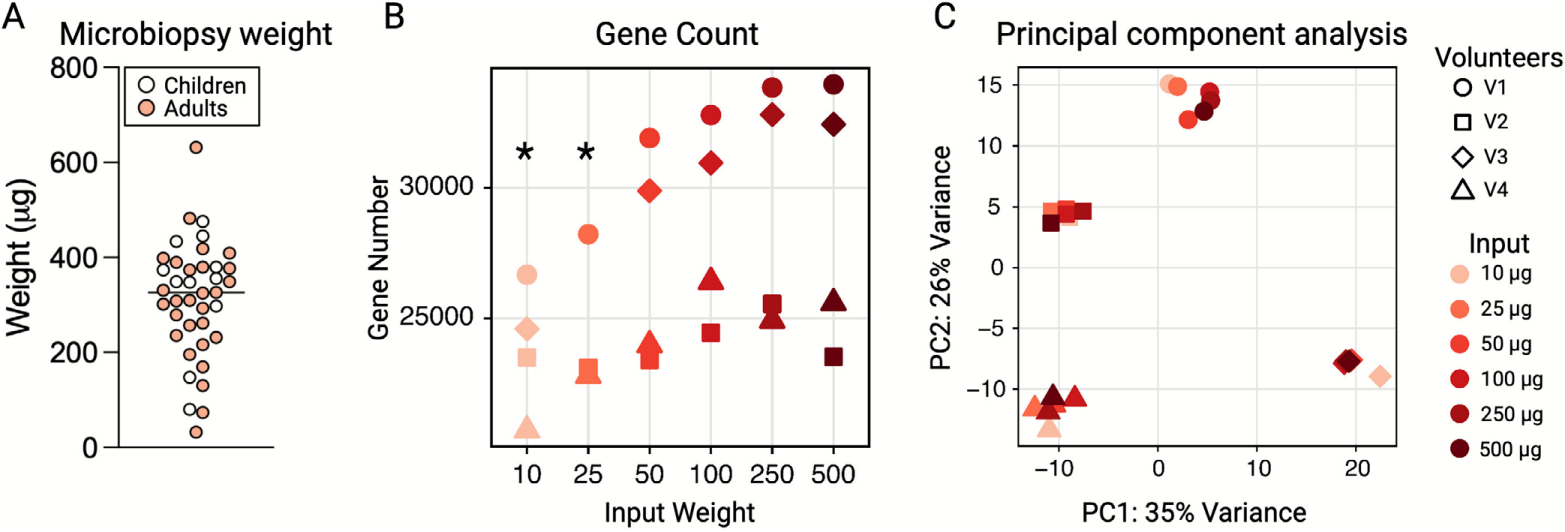
Sample yield and analyzability of microbiopsies. (A) Weights of freeze‐dried microbiopsy samples. Dots represent individual values. (B) Number of genes found in sequenced muscle samples of different input weights. * = *p* < 0.05 versus 100 μg, 250 μg, and 500 μg. (C) Principal component analysis of gene expression for all samples.

The average material yield from the microbiopsies was 312.1 μg (SD 121.8 μg) (freeze‐dried weight; Figure 2A). Three of the 39 measured samples weighed less than 100 μg, at 32.5, 73.4, and 80.2 μg, respectively. No difference in mean weight was observed for biopsies taken from children or adults (335.0 ± 121.3 μg vs. 303.1 ± 122.9 μg, *p* = 0.468). Freeze‐dried tissue samples from the Bergström biopsies from four participants were used to create a muscle tissue input ladder (10–25–50–100–250–500 μg). Using RNA‐seq, we found that the average number of genes detected at each input weight ranged from 23,863 ± 2485 to 29,227 ± 4699. Fewer detectable genes were found in the samples using input weights of 10 and 25 μg, compared to 100, 250, and 500 μg (*p* < 0.05; Figure 2B). A principal component analysis showed that the input weights did not significantly affect the transcriptomic signature of each sample (Figure 2C).

## 4 Discussion

Our main findings suggest that the 20‐gage microbiopsy procedure is well‐tolerated in both children and adults and that the tissue yield from a single biopsy is sufficient to accurately measure the transcriptomic signature of skeletal muscle tissue.

The children rated the perceived pain of one microbiopsy as relatively low, indicating an overall tolerable procedure. This was, however, significantly more painful than venipuncture, although whether this is clinically significant is uncertain. Venipuncture is a widely used and accepted procedure in clinical pediatric practice; hence, we consider venipuncture to be a suitable procedure to which to compare our microbiopsy data. The pain estimate for venipuncture was lower than that observed in previous studies. For example, children aged 6–12 years rated VAS as 5.2 (2.8 SD) [17] and 2 (IQR: 2) [18], respectively, for venipuncture without local anesthetic cream. In the present study as opposed to the previously mentioned study, prior application of local anesthetics likely contributed to the nearly pain‐free experience of venipuncture. Evaluating perceived pain is inherently complex and factors extend beyond the interpretation of physical injury (pain) to also involve psychological anticipation (fear) [19]; the use of music in combination with an experienced nurse in the present study may have further contributed to the low pain ratings. Prior publications on the tolerability of the microbiopsy procedure in children are sparse. Deschrevel and coworkers included children aged 2–9 years and sampled muscle from the medial gastrocnemius and the semitendinosus. The authors assessed perceived pain postsurgery using a Wong‐Baker faces pain rating scale, with ~50% of the children reporting little to no pain, ~30% moderate pain and ~20% severe to very severe pain in the hours following surgery [20]. However, due to the biopsies being taken during surgery, pain could not be assessed in conjunction with the biopsy procedure.

We found that the perceived pain of the microbiopsy was similar for children and adults. The adults rated microbiopsy as less painful than both the Bergström biopsy and the prior application of infiltrative local anesthetic, albeit with a low absolute difference. This is in line with previous work, in which Bonafiglia and collaborators showed that a microbiopsy with an approximately 2 mm needle and prior subcutaneous local anesthetic was perceived as significantly less painful compared to the Bergström biopsy [21]. Somewhat contrasting to the results presented in this study, Tobina and coworkers reported that a 16‐gage needle biopsy with prior application of local anesthetic cream rendered similar perceived pain to that of venipuncture without prior local anesthetic [13]. However, it is likely that the contrasting findings are due to the low pain scores observed within our data for venipuncture, rather than a large difference in the reported pain of the microbiopsy procedure.

The analyzability of larger 14–16‐gage microbiopsy samples has been explored in various downstream applications. However, most studies using these larger needles still employ multiple sequential biopsy samplings to ensure sufficient material yield before validation. They have demonstrated the successful use of the microbiopsy procedure for downstream gene and protein expression analyses via RT‐PCR and Western blot [13], enzyme activity assays [8], and mitochondrial respiration assessments [22]. Adding to this body of work, we used our muscle tissue input ladder to demonstrate that the transcriptomic profile of skeletal muscle could be accurately characterized with as little as 10 μg of freeze‐dried tissue, suggesting that a single 20‐gage microbiopsy typically provides sufficient material for RNA‐seq. This highlights the potential of the 20‐gage microbiopsy needle technique to generate novel omics data on skeletal muscle, particularly in healthy pediatric populations. That would allow for insight into the temporal response of skeletal muscles in children following varying stimuli (e.g., exercise interventions).

The utility of 20‐gage microbiopsy samples for histological or immunohistochemical analyses remains to be determined. Several studies have reported sufficient material for muscle cross‐sectional analysis using 16‐gage microbiopsy needles [8, 20, 21, 22]. However, given that 16‐gage microbiopsy yields approximately 5–15 times more tissue per biopsy [8, 13, 22] than from the 20‐gage microbiopsy reported here, the suitability of the latter for histological assessments remains questionable. Pertaining to clinical implications, further validation is required; however, microbiopsies could provide a low‐threshold insight into the transcriptome and potentially other aspects of skeletal muscle biology.

### 4.1 Limitations

Rating pain is inherently difficult and is likely influenced by a multitude of factors beyond nociceptive stimuli. Potentially, factors such as cultural context may affect the results, warranting caution when generalizing findings to other settings. Upon failure to collect muscle tissue, a repeated biopsy was performed, and perceived pain was rated for this biopsy and used for analysis. The evaluation of RNA‐seq was conducted on material sampled using the Bergström biopsy. While the microbiopsy yield was well within the evaluated range, RNA‐seq has yet to be performed on samples obtained via a 20‐gage microbiopsy.

## 5 Conclusion

The microbiopsy procedure represents a less painful method for muscle tissue sampling, rendering sufficient yield to perform RNA sequencing with reliable outcomes. This underscores the potential of the 20‐gage microbiopsy as a valuable tool for muscle research, particularly in nonclinical settings and pediatric populations, for which minimizing procedural burden is a key consideration.

## Author Contributions

**Emil Rydell Högelin** and **Sebastian Edman:** writing – original draft. **Emil Rydell Högelin**, **Sebastian Edman**, **Paulo R. Jannig**, **Axel Löfgren**, **Kajsa Thulin**, **Piotr Michno**, **Jessica Norrbom**, **Björn A. Alkner**, **Ferdinand von Walden**, and **Lotta Fornander:** writing – reviewing and editing. **Emil Rydell Högelin**, **Sebastian Edman**, **Kajsa Thulin**, **Piotr Michno**, **Jessica Norrbom**, **Björn A. Alkner**, **Ferdinand von Walden**, and **Lotta Fornander:** conceptualization. **Emil Rydell Högelin**, **Axel Löfgren**, **Kajsa Thulin**, **Piotr Michno**, **Jessica Norrbom**, **Björn A. Alkner**, **Ferdinand von Walden**, and **Lotta Fornander:** data collection. **Emil Rydell Högelin**, **Sebastian Edman**, and **Paulo R. Jannig:** data analysis. **Emil Rydell Högelin**, **Sebastian Edman**, **Ferdinand von Walden**, and **Lotta Fornander:** data interpretation. **Emil Rydell Högelin**, **Sebastian Edman**, and **Paulo R. Jannig:** data visualization. **Emil Rydell Högelin**, **Sebastian Edman**, **Kajsa Thulin**, **Jessica Norrbom**, **Björn A. Alkner**, **Ferdinand von Walden**, and **Lotta Fornander:** funding acquisition.

## Funding

This study was funded by the Futurum—The Academy for Health and Care, Region Jönköping County (FUTURUM‐964488) awarded to K.T., E.R.H., P.M., and B.A. S.E. was supported by grants from Linnea och Josef Carlssons Stiftelse, Stiftelsen Sunnerdahls Handikappfond, KI Research Foundation Grants (#2024‐02598), Stiftelsen Frimurare Barnhuset i Stockholm, Sällskapet Barnavård, Kronprinsessan Lovisas Förening för Barnasjukvård, O.E. och Edla Johanssons Vetenskapliga Stiftelse, and Svenska Sällskapet för Medicinsk Forskning (PG‐24‐0429). J.N. was supported by the Swedish Research Council for Sport Science (P2024‐0119) and the Promobilia Foundation (S24502). F.v.W. was supported by The Swedish Research Council (2022‐01392), Neuroförbundet (SB2025‐0005), Svenska Sällskapet för Medicinsk Forskning (SG‐25‐0199‐B‐H‐01), and the Promobilia Foundation (S24502).

## Ethics Statement

We confirm that we have read the Journal's position on issues involved in ethical publication and affirm that this report is consistent with those guidelines.

## Conflicts of Interest

The authors declare no conflicts of interest.

## Data Availability

RNA‐sequencing processed data and analysis code are available on Zenodo (https://doi.org/10.5281/zenodo.17494250 ) and GitHub (https://github.com/paulojannig/Hogelin_Edman_et_al_Microbiopsy). Raw data will be made available upon request.
